# A Public Database of Immersive VR Videos with Corresponding Ratings of Arousal, Valence, and Correlations between Head Movements and Self Report Measures

**DOI:** 10.3389/fpsyg.2017.02116

**Published:** 2017-12-05

**Authors:** Benjamin J. Li, Jeremy N. Bailenson, Adam Pines, Walter J. Greenleaf, Leanne M. Williams

**Affiliations:** ^1^Department of Communication, Stanford University, Stanford, CA, United States; ^2^Department of Psychiatry and Behavioral Sciences, School of Medicine, Stanford University, Stanford, CA, United States

**Keywords:** virtual reality, database, immersive VR clips, head movement, affective ratings

## Abstract

Virtual reality (VR) has been proposed as a methodological tool to study the basic science of psychology and other fields. One key advantage of VR is that sharing of virtual content can lead to more robust replication and representative sampling. A database of standardized content will help fulfill this vision. There are two objectives to this study. First, we seek to establish and allow public access to a database of immersive VR video clips that can act as a potential resource for studies on emotion induction using virtual reality. Second, given the large sample size of participants needed to get reliable valence and arousal ratings for our video, we were able to explore the possible links between the head movements of the observer and the emotions he or she feels while viewing immersive VR. To accomplish our goals, we sourced for and tested 73 immersive VR clips which participants rated on valence and arousal dimensions using self-assessment manikins. We also tracked participants' rotational head movements as they watched the clips, allowing us to correlate head movements and affect. Based on past research, we predicted relationships between the standard deviation of head yaw and valence and arousal ratings. Results showed that the stimuli varied reasonably well along the dimensions of valence and arousal, with a slight underrepresentation of clips that are of negative valence and highly arousing. The standard deviation of yaw positively correlated with valence, while a significant positive relationship was found between head pitch and arousal. The immersive VR clips tested are available online as supplemental material.

## Introduction

Blascovich et al. (2002) proposed the use of virtual reality (VR) as a methodological tool to study the basic science of psychology and other fields. Since then, there has been a steady increase in the number of studies that seek to use VR as a tool (Schultheis and Rizzo, 2001; Fox et al., 2009). Some studies use VR to examine how humans respond to virtual social interactions (Dyck et al., 2008; Schroeder, 2012; Qu et al., 2014) or as a tool for exposure therapy (Difede and Hoffman, 2002; Klinger et al., 2005), while others employ VR to study phenomenon that might otherwise be impossible to recreate or manipulate in real life (Slater et al., 2006; Peck et al., 2013). In recent years, the cost of a typical hardware setup has decreased dramatically, allowing researchers to spend less than the typical price of a laptop to implement compelling VR. One of the key advantages of VR for the study of social science is that sharing of virtual content will allow “not only for cross-sectional replication but also for more representative sampling” (Blascovich et al., 2002). What is needed to fulfill this vision is a database of standardized content.

The immersive video (or immersive VR clip) is one powerful and realistic aspect of VR. It shows a photorealistic video of a scene that updates based on head-orientation but is not otherwise interactive (Slater and Sanchez-Vives, 2016). When a viewer watches an immersive VR clip, he sees a 360° view from where the video was originally recorded, and while changes in head orientation are rendered accurately, typically these videos do not allow for head translation. A video is recorded using multiple cameras and stitched together through software to form a total surround scene. In this sense, creating content for immersive video is fairly straightforward, and consequently there is a wealth of content publicly available on social media sites (Multisilta, 2014).

To accomplish the goal of a VR content database, we sourced and created a library of immersive VR clips that can act as a resource for scholars, paralleling the design used in prior studies on affective picture viewing (e.g., International Affective Picture System, IAPS; Lang et al., 2008). The IAPS is a large set of photographs developed to provide emotional stimuli for psychological and behavioral studies on emotion and mood induction. Participants are shown photographs and asked to rate each on the dimensions of valence and arousal. While the IAPS and its acoustic stimuli counterpart the International Affective Digital Sounds (IADS; Bradley and Lang, 1999) are well-established and used extensively in emotional research, a database of immersive VR content that can potentially induce emotions does not exist to our knowledge. As such, we were interested to explore if we can establish a database of immersive VR clips for emotion induction based on the affective response of participants.

Most VR systems allow a user to have a full 360° head rotation view, such that the content updates based on the particular orientation of the head. In this sense, the so-called field of regard is higher in VR than in traditional media such as the television, which doesn't change when one moves her head away from the screen. This often allows VR to trigger strong emotions in individuals (Riva et al., 2007; Parsons and Rizzo, 2008). However, few studies have examined the relationship between head movements in VR and emotions. Darwin (1965) discussed the idea of head postures representing emotional states. When one is happy, he holds his head up high. Conversely, when he is sad, his head tends to hang low. Indeed, more recent empirical research has provided empirical evidence for these relationships (Schouwstra and Hoogstraten, 1995; Wallbott, 1998; Tracy and Matsumoto, 2008).

An early study which investigated the influence of body movements on presence in virtual environments found a significant positive association between head yaw and reported presence (Slater et al., 1998). In a study on head movements in VR, participants saw themselves in a virtual classroom and participated in a learning experience (Won et al., 2016). Results showed a relationship between lateral head rotations and anxiety, where the standard deviation of head yaw significantly correlated to the awareness and concern individuals had regarding other virtual people in the room. Livingstone and Palmer (2016) tasked vocalists to speak and sing passages of varying emotions (e.g., happy, neutral, sad) and tracked their head movements using motion capture technology. Findings revealed a significant relationship between head pitch and emotions. Participants raised their heads when vocalizing passages that conveyed happiness and excitement and lowered their heads for those of a sad nature. Understanding the link between head movements in VR and emotions may be key in the development and implementation of VR in the study and treatment of psychological disorders (Wiederhold and Wiederhold, 2005; Parsons et al., 2007).

There are two objectives of the study: First, we seek to establish and allow public access to a database of immersive VR clips that can act as a potential resource for studies on emotion induction using virtual reality. Second, given we need a large sample size of participants to get reliable valence and arousal ratings for our video, we are in a unique position explore the possible links between head movements and the emotions one feels while viewing immersive VR. To accomplish our goals, we sourced for and tested 73 immersive VR clips which participants rated on valence and arousal dimensions using self-assessment manikins. These clips are available online as supplemental material. We also tracked participants' rotational head movements as they watched the clips, allowing us to correlate the observers' head movements and affect. Based on past research (Won et al., 2016), we predicted significant relationships between the standard deviation of head yaw with valence and arousal ratings.

## Methods

### Participants

Participants comprised of undergraduates from a medium-sized West Coast university who received course credit for their participation. In total, 95 participants (56 female) between the ages of 18 and 24 took part in the study.

### Stimulus and measures

The authors spent 6 months searching for clips of immersive VR which they thought will effectively induce emotions. Sources include personal contacts and internet searches on website such as *YouTube, Vrideo*, and *Facebook*. In total, more than 200 immersive VR clips were viewed and assessed. From this collection, 113 were shortlisted and subjected to further analysis. The experimenters evaluated the video clips and a subsequent round of selection was conducted based on the criteria employed by Gross and Levenson (1995). First, the clips had to be of relatively short length. This is especially important as longer clips may induce fatigue and nausea among participants. Second, the VR clips had to be understandable on their own without the need for further explanation. As such, clips which were sequels or part of an episodic series were excluded. Third, the VR clips should be likely to induce valence and arousal. The aim is to get a good spread of videos that will vary across the dimensions. A final 73 immersive VR clips were selected for the study. They ranged from 29 to 668 s in length with an average of 188 s per clip.

**Figure 1:**
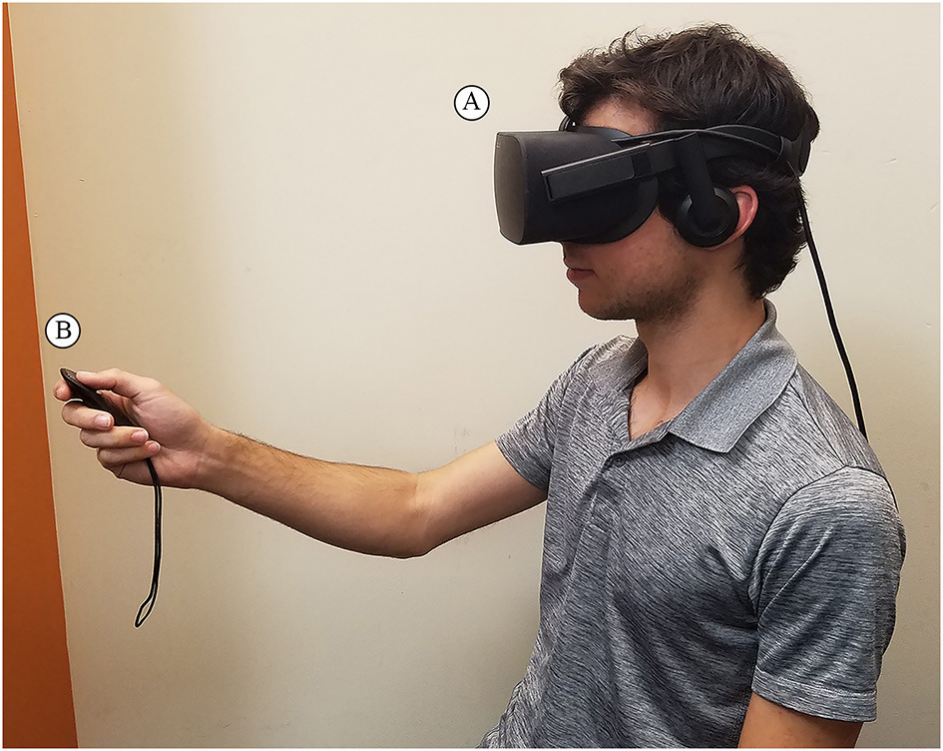
The experimental setup depicting a participant **(A)** wearing an Oculus Rift HMD to view the immersive VR clips, and **(B)** holding an Oculus. Rift remote to select his affective responses to his viewing experience.

Participants viewed the immersive VR clips through an *Oculus Rift* CV1 (Oculus VR, Menlo Park, CA) head-mounted display (HMD). The *Oculus Rift* has a resolution of 2,160 × 1,200 pixels, a 110° field of view and a refresh rate of 90 Hz. The low-latency tracking technology determines the relative position of the viewer's head and adjusts his view of the immersive video accordingly. Participants interacted with on-screen prompts and rated the videos using an *Oculus Rift* remote. *Vizard* 5 software (Worldviz, San Francisco, CA) was used to program the rating system. The software ran on a 3.6 GHz Intel i7 computer with an *Nvidia* GTX 1080 graphics card. The experimental setup is shown in Figure 1.

**Figure 2:**
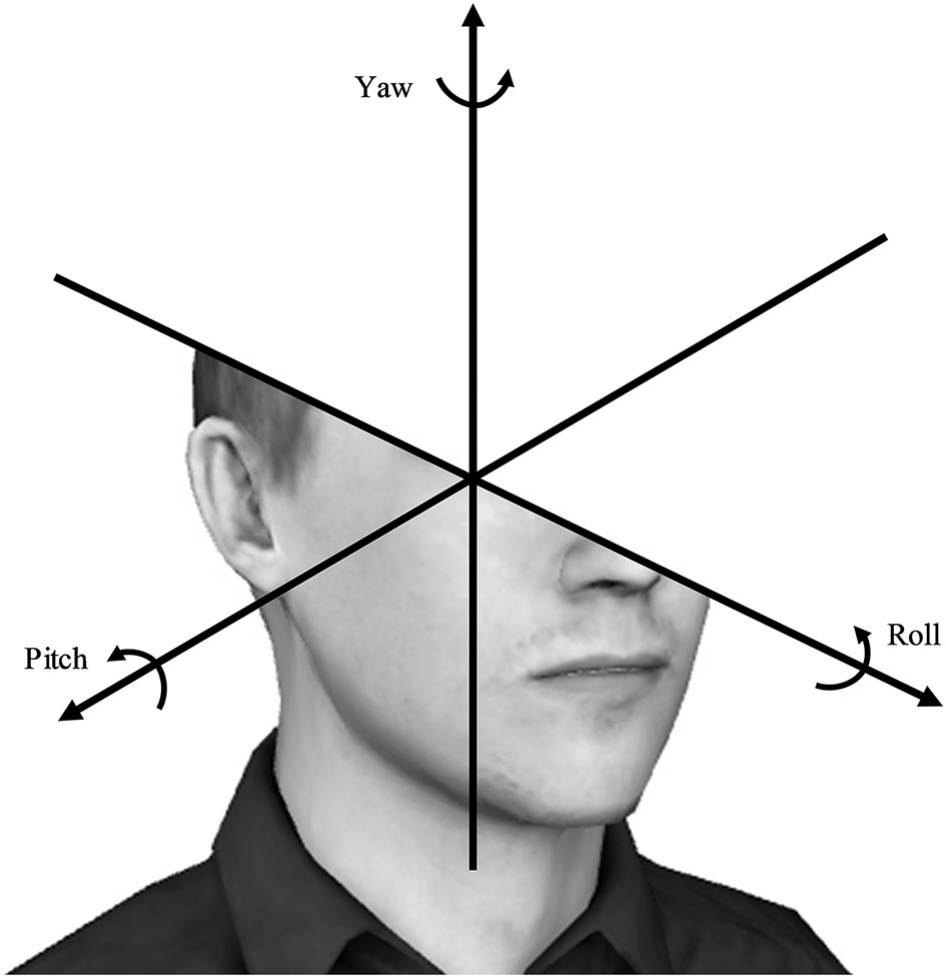
Depiction of the three angles of rotational movement- pitch, yaw, and roll.

The *Oculus Rift* HMD features a magnetometer, gyroscope, and accelerometer which combine to allow for tracking of rotational head movement. The data was digitally captured and comprised of the pitch, yaw, and roll of the head. These are standard terms for rotations around the respective axes, and are measured in degrees. Pitch refers to the movement of the head around the X-axis, similar to a nodding movement. Yaw represents the movement of the head around the Y-axis, similar to turning the head side-to-side to indicate “no.” Roll refers to moving the head around the Z-axis, similar to tilting the head from one shoulder to the other. These movements are presented in Figure 2. As discussed earlier, Won et al. (2016) found a relationship between lateral head rotations and anxiety. They showed that scanning behavior, defined as the standard deviation of head yaw, significantly correlated with the awareness and concern people had of virtual others. In this study, we similarly assessed how much participants moved their heads by calculating the standard deviations of the pitch, yaw, and roll of their head movements while they watched each clip and included them as our variables.

**Figure 3:**
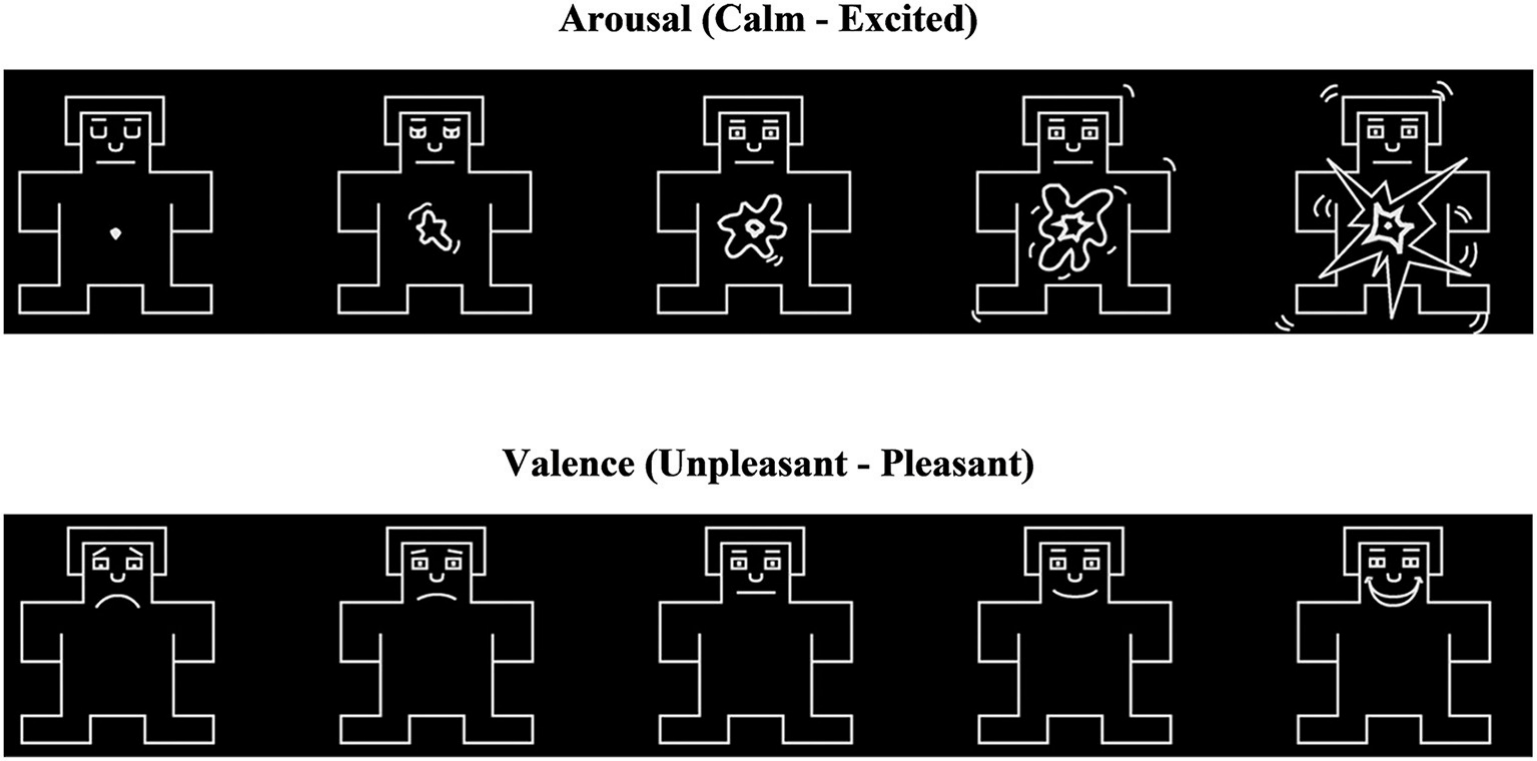
SAM figures to measure valence and arousal ratings.

Participants made their ratings using the self-assessment manikin (SAM; Lang, 1980). SAM shows a series of graphical figures that range along the dimensions of valence and arousal. The expressions of these figures vary across a continuous scale. The SAM scale for valence shows a sad and unhappy figure on one end, and a smiling and happy figure at the other. For arousal, the SAM scale depicts a calm and relaxed figure on one end, and an excited and interested figure on the other. A 9-point rating scale is presented at the bottom of each SAM. Participants select one of the options while wearing the HMD using the *Oculus Rift* remote control device that could scroll among options. Studies have shown that SAM ratings of valence and arousal are similar to those obtained from the verbal semantic differential scale (Lang, 1980; Ito et al., 1998). The SAM figures are presented in Figure 3.

### Procedure

Pretests were conducted to find out the duration that participants were comfortable with watching immersive videos before they experience fatigue or simulation sickness. Results revealed that some participants encountered fatigue and/or nausea if they watched for more than 15 min without a break. Most participants were at ease with a duration of around 12 min. The 73 immersive VR clips were then divided into clusters with an approximate duration of 12 min per cluster. This resulted in a total of 19 groups of videos. Based on the judgment of the experimenters, no more than two clips of a particular valence (negative/positive) or arousal (low/high) were shown consecutively (Gross and Levenson, 1995). This was to discourage participants from being too involved in any particular affect and influence his judgement in the subsequent ratings. Each video clip was viewed by a minimum of 15 participants.

When participants first arrived, they were briefed by the experimenter that the purpose of the study was to examine how people respond to immersive videos. Participants were told that they would be wearing an HMD to view the immersive videos, and that they can request to stop participating at any time if they feel discomfort, nauseous, or some form of simulator sickness. Participants were then presented with a printout of the SAM measures for valence and arousal, and told that they would be rating the immersive videos based on these dimensions. Participants were then introduced to the *Oculus Rift* remote and its operation in order to rate the immersive VR clips.

The specific procedure is presented here: Participants sat on swivel chair which allowed them to turn around 360° if they wished to. They first watched a test immersive VR clip and did a mock rating to get accustomed to the viewing and rating process. They then watched and rated a total of three groups of video clips with each group comprising of between two and four video clips. A 5 s preparation screen was presented before each clip. After the clip was shown, participants were presented with the SAM scale for valence. After participants selected the corresponding rating using the *Oculus Rift* remote, the SAM scale for arousal was presented and participants made their ratings. Following this, the aforementioned 5 s preparation screen was presented to get participants ready to view the next clip. After watching one group of immersive VR clips, participants were given a short break of about 5 min before continuing with the next group of clips. This was done to minimize the chances of participants feeling fatigue or nauseous by allowing them to rest in between group of videos. With each group of videos having a duration of about 12 min, the entire rating process lasted around 40 min.

## Results

### Affective ratings

**Figure 4:**
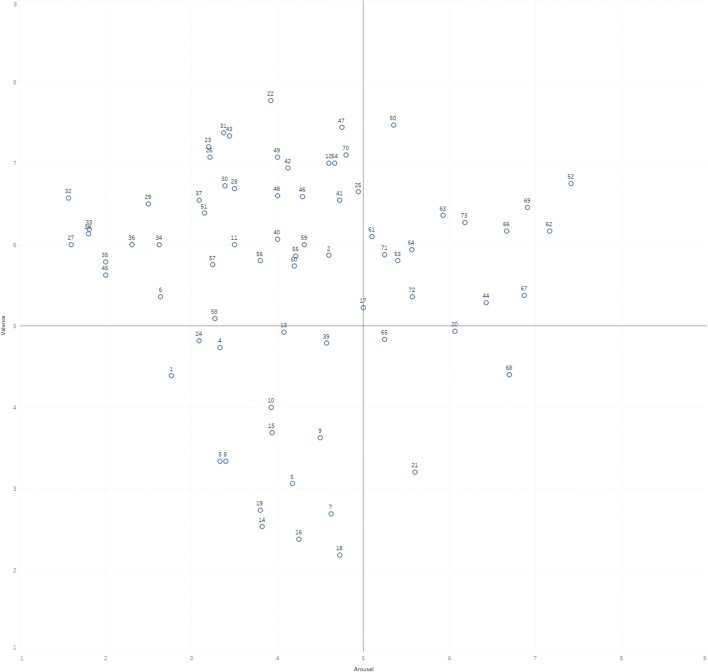
Scatterplot of immnersive video clips defined by mean arousal and valence ratings.

Figure 4 shows the plots of the immersive video clips (labeled by their ID numbers) based on mean ratings of valence and arousal. There is a varied distribution of video clips above the midpoint (5) of valence that vary across arousal ratings. However, despite our efforts to locate and shortlist immersive VR clips for the study, there appears to be an underrepresentation for clips that both induce negative valence and are highly arousing. Table 1 shows a list of all the clips in the database, together with a short description, length and their corresponding valence and arousal ratings.

**Table 1:** Comprehensive list of all immersive VR clips in database.

**ID**	**Title**	**Description**	**Length (s)**	**Valence**	**Arousal**
1	Abandoned building	Daytime shot of an alley in between two abandoned buildings, with ambient music	120	4.39	2.77
2	A Mumbai summer	Tour of the Mumbai, India, various shots of urban and suburban locations	199	5.87	4.60
3	Abandoned city	Virtual environment of a post-apocalyptic abandoned city	50	3.33	3.33
4	Alaska's melting glaciers	Educational clip about the effects of climate change on Alaska's glaciers	234	4.73	3.33
5	Chernobyl	Educational clip on the effects of the Chernobyl nuclear disaster on the town of Pripyat	548	3.06	4.18
6	Sadness elicitation—lake valley	Virtual environment of a desolate valley	119	5.36	2.64
7	Fukushima	Journalistic feature on the effects of the Fukushima nuclear crisis	560	2.69	4.63
8	Happyland	Short documentary on a Manila dumpsite where 40,000 people call home	611	3.33	3.40
9	Homeless veterans	Educational clip on the plight of US veterans who are homeless	164	3.63	4.50
10	New York 2121	Virtual environment of a post-apocalyptic and abandoned New York City	120	4.00	3.93
11	North Korea	Journalistic clip on the everyday life of North Koreans	640	6.00	3.50
12	The fight to save threatened species	Various shots of animals threatened by extinction	124	7.00	4.60
13	The margins	Journalistic clip on illegal border crossing	137	4.92	4.08
14	War zone	Journalistic clip of a war torn city	183	2.53	3.82
15	Inside a bee hive	The clip gives the viewer an upclose view of the inside of a bee hive together with a large number of bees	43	3.69	3.94
16	Solitary confinement	Short film on the emotional trauma of solitary confinement	221	2.38	4.25
17	Survive a bear attack	Short film on campers handling a bear attack	90	5.22	5.00
18	The displaced	Journalistic feature on three homeless children	668	2.18	4.73
19	The Nepal earthquake aftermath	Short film on the effects of an earthquake in Nepal	240	2.73	3.80
20	War knows no nation	Short filmo on war that mixes computer-graphics with live-action footage	448	4.93	6.07
21	Zombie apocalypse horror	Action film where the viewer follows a group of soldiers defending against a zombie attack	265	3.20	5.60
22	Great ocean road	Aerial shots over various scenic locations in Australia	118	7.77	3.92
23	Instant caribbean vacation	Promotional video of a Caribbean cruise liner	150	7.20	3.20
24	Blyde canyon	Promotional video introducing the features and scenic views of a large canyon	157	4.82	3.09
25	The most beautiful place in the world	Various scenic shots of a man's travels	186	6.65	4.94
26	Getting licked by a cow in Ireland	Viewer gets a closeup experience with a cow	65	7.07	3.21
27	Seagulls	Various video clips of seagulls at a quiet seaside	120	6.00	1.60
28	Maldives beach and resort	Various clips filmed at a beach in Maldives	138	6.69	3.50
29	Fallen Trees	Atmospheric clip of various fallen trees in the forest	146	6.50	2.50
30	Haleakala national park sunrise	Timelapse clip showing the sun rising over a forest	37	6.72	3.39
31	Ibiza wedding	Various shots of a couple's wedding party held at a beach resort	310	7.38	3.38
32	Malaekahana sunrise	Viewer sees the sun rising over the horizon at a beach	120	6.57	1.57
33	Pacific sunset half moon bay	Timelapse clip showing the sunset close to the sea	134	6.19	1.81
34	Raising ducklings	Viewer gets a closeup experience with ducklings	203	6.00	2.63
35	Redwoods: Walk Among Giants	Various landscape shots of tall trees in forest	120	5.79	2.00
36	Rocky beach	Scenic shots of a beach	93	6.00	2.31
37	Sunset of oia-santorini	Timelapse clip of sunset over a Grecian town	89	6.55	3.09
38	Mountain stillness	Atmospheric shots of Canadian snowy mountains	128	6.13	1.80
39	Zip-lining in chattanooga	Viewer takes the perspective of a zip-liner in action	127	4.79	4.57
40	VR kittens	Various up close shots of kittens	101	6.07	4.00
41	Fighter jet patrouile suisse	Viewer takes the perspective of a fighter jet pilot in command of an airplane	120	6.55	4.73
42	Cute kittens battle	Video clip showing four kittens playing with one another	65	6.94	4.13
43	Alice the first Swedish baby goes VR	Various shots of an infant in outdoor environments	126	7.33	3.44
44	Conquer the mega ramp	Viewer takes the perspective of an extreme sports participant and goes down a huge slope before leaping across a gap	86	5.29	6.43
45	Joy elicitation	Virtual environment of a field with flowers and butterflies	119	5.63	2.00
46	Explore the world with IM360	Various shots of popular tourist destinations	197	6.59	4.29
47	Puppy Bowl XII	Viewers watch puppies compete in a mock football match	192	7.44	4.75
48	Holi festival of colors	Journalistic clip on a popular festival in India	173	6.60	4.00
49	India's first ever 360 Wedding Video	Various shots at an Indian wedding	201	7.07	4.00
50	Puppies host SourceFed for a day	Viewers get up close with some puppies	80	7.47	5.35
51	Resonance: a jump VR Video	An experimental film that follows the journeys of a violin player	275	6.39	3.15
52	Speed flying	Viewer follows a speed wing pilot as he glides past mountains	154	6.75	7.42
53	Tomorrowland 2014	A highlights reel of the events at a popular music festival	265	5.80	5.40
54	As It Is	A trailer for a documentary on the history of the Grand Canyon	154	7.00	4.67
55	New York City Jump	Journalistic clip on the popular spots in New York City	144	5.86	4.21
56	Solar impulse assembles the mobile hangar	A time lapse clip on the setting up of a temporary airplane hangar	129	5.80	3.80
57	Les berges du center à Wasquehal	A promotional clip for a condominium, where viewers get to explore the features and interior	87	5.75	3.25
58	Spangler Lawn	A view of people spending an afternoon relaxing in a courtyard	58	5.09	3.27
59	Seeking Pluto's Frigid Heart	A journalistic clip on the features of the planet Pluto	463	6.00	4.31
60	Russian knights acrobatic rehearsals	Viewer takes the perspective of a fighter jet pilot involved in airshow rehearsals	120	5.73	4.20
61	Kodak SP360 Yacht	A view of a yacht out at sea	29	6.10	5.10
62	Mega Coaster	Viewer takes the perspective of an extreme sports participant leaping off a ramp	117	6.17	7.17
63	NASA: Encapsulation & Launch of OSIRIS Rex	Documentary film on the planning and execution of rocket launches	285	6.36	5.93
64	Surrounded by elephants	Viewer has an up close experience with elephants in a field	156	5.94	5.56
65	Kidnapped	A short comedy where viewer takes the perspective of a kidnap victim in a case of mistaken identity	406	4.83	5.25
66	Great Hammerhead Shark Encounter	Viewer gets an up close experience with sharks in the sea	134	6.17	6.67
67	Canyon Swing	Viewer experiences swinging over an open canyon	104	5.38	6.88
68	Jailbreak 360	Short action film depicting a jailbreak from various closed-circuit cameras and how the culprit was captured	339	4.40	6.70
69	Walk the tight rope	Viewer experiences walking a tight rope over a canyon	151	6.46	6.91
70	Tahiti Surf	Viewer experiences snorkeling and surfing on a Tahitian beach	205	7.10	4.80
71	Lion's Last Stand	Viewer gets an up close experience with a tiger on a savanna	40	5.88	5.25
72	Relive Undertaker's Entrance	Viewer experiences a sports entertainment event at a packed stadium	122	5.36	5.57
73	Through Mowgli's Eyes	A short film where the viewer observes a conversation between an ape and a boy	93	6.27	6.18

The immersive VR clips varied on arousal ratings (*M* = 4.20, *SD* = 1.39), ranging from a low of 1.57 to a high of 7.4. This compares favorably with arousal ratings on the IAPS, which range from 1.72 to 7.35 (Lang et al., 2008). Comparatively, arousal ratings on the IAPS ranged from 1.72 to 7.35. The video clips also varied on valence ratings (*M* = 5.59, *SD* = 1.40), with a low of 2.2 and a high of 7.7. This compares reasonably well with valence ratings on the IAPS, which range from 1.31 to 8.34.

### Head movement data

**Figure 5:**
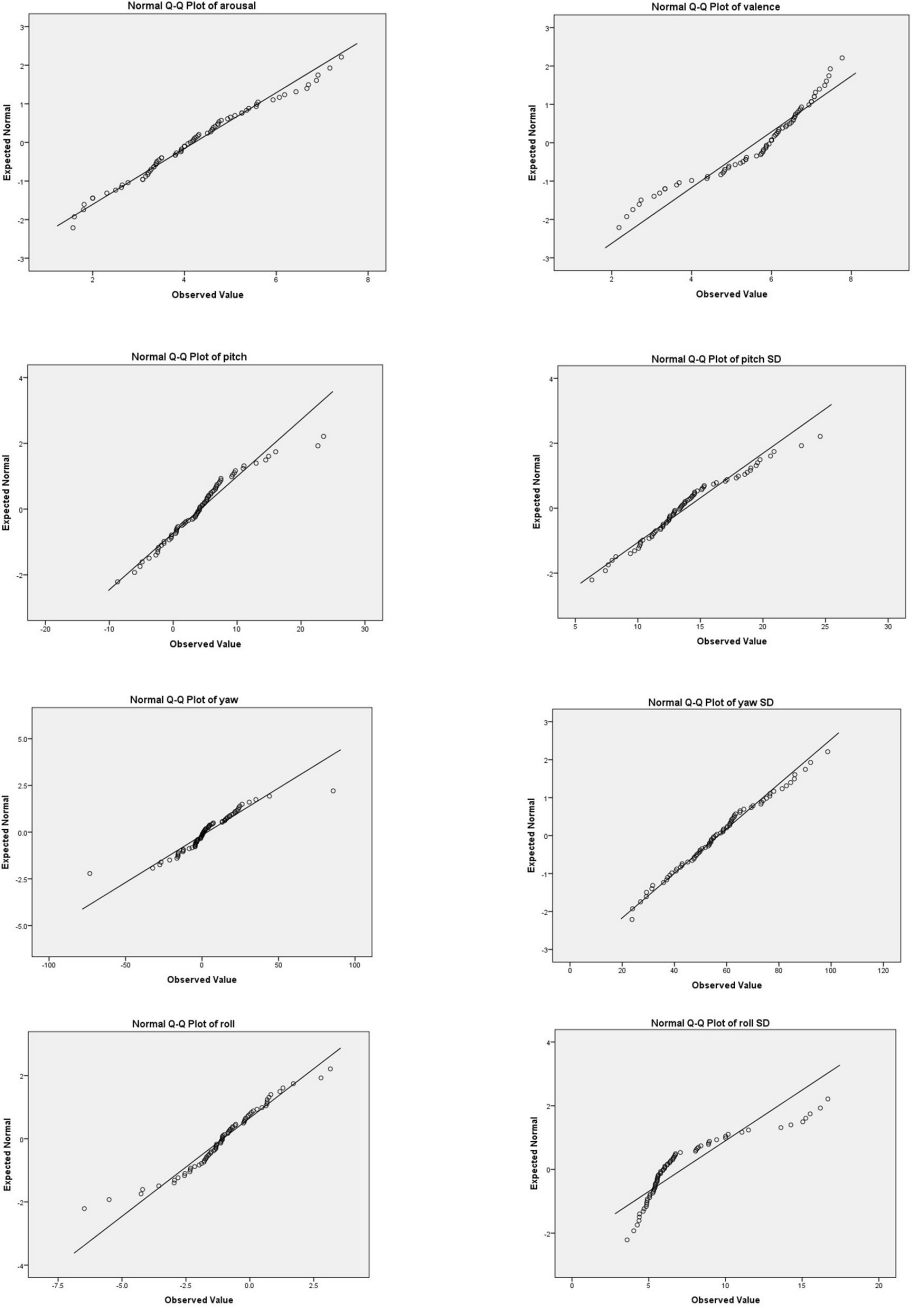
Normal Q-Q plots of all observed variables.

**Figure 6:**
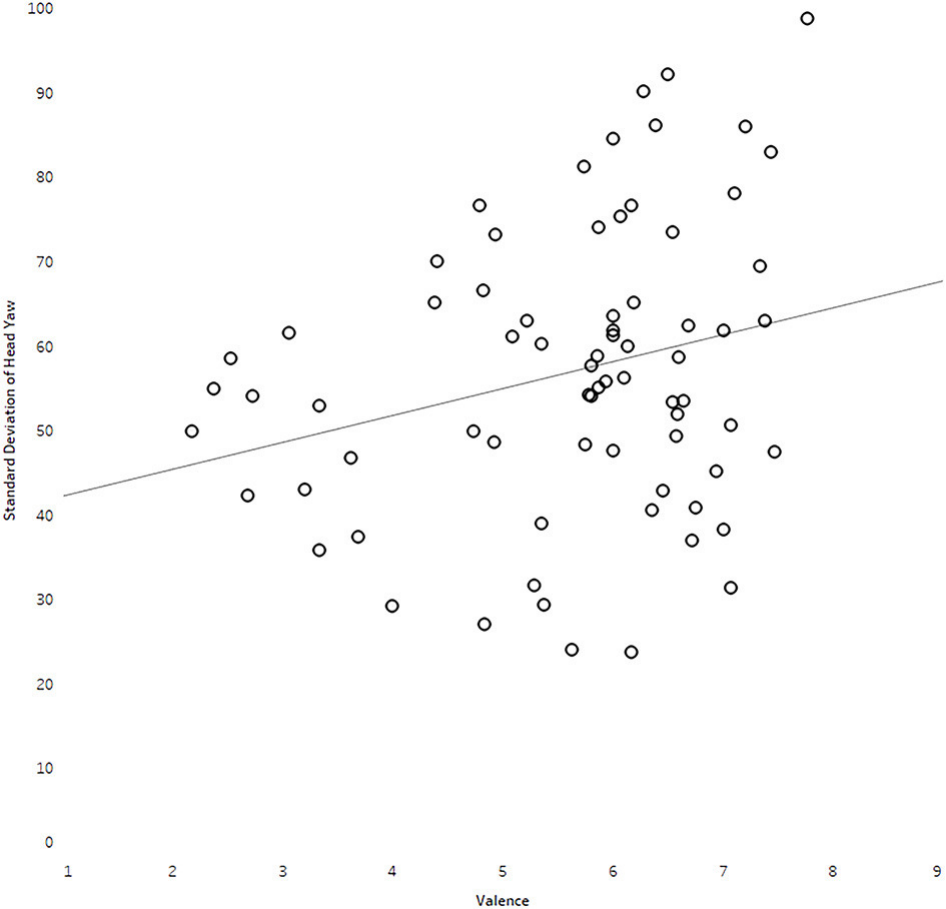
Plot illustrating relationship between standard deviation of head yaw and valence ratings.

**Figure 7:**
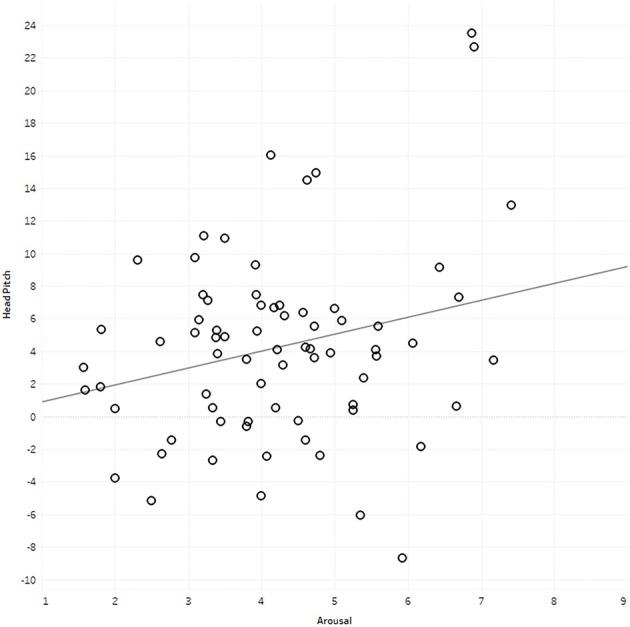
Plot illustrating the relationship between head pitch and arousal ratings.

Pearson's product-moment correlations between observers' head movement data and their affective ratings are presented in Table 2. Most scores appear to be normally distributed as assessed by a visual inspection of Normal Q-Q plots (see Figure 5). Analyses showed that average standard deviation of head yaw significantly predicted valence [*F*_(1, 71)_ = 5.06, *p* = 0.03, *r* = 0.26, adjusted *R*^2^ = 0.05], although the direction was in contrast to our hypothesis. There was no significant relationship between standard deviation of head yaw with arousal [*F*_(1, 71)_ = 2.02, *p* = 0.16, *r* = 0.17, adjusted *R*^2^ = 0.01)]. However, there was a significant relationship between average head pitch movement and arousal [*F*_(1, 71)_ = 4.63, *p* = 0.04, *r* = 0.25; adjusted *R*^2^ = 0.05]. Assumptions of the *F*-test for the significant relationships were met, with analyses showing homoscedasticity and normality of the residuals. The plots of the significant relationships are presented in Figures 6, 7.

**Table 2:** Correlation matrix of head movement data and affective ratings (*N* = 73).

	**Valence**	**Arousal**
Pitch (*M* = 4.23, *SD* = 5.86)	0.02	0.25^*^
Pitch Standard Deviation (*M* = 13.90, *SD* = 3.64)	0.16	0.10
Yaw (*M* = 3.67, *SD* = 20.25)	0.0	0.08
Yaw Standard Deviation (*M* = 57.14, *SD* = 16.96)	0.27^*^	−0.17
Roll (*M* = −1.11, *SD* = 1.65)	0.20	−0.19
Roll Standard Deviation (*M* = 7.32, *SD* = 3.47)	0.08	−0.02

* *p < 0.05*.

## Discussion

The first objective of the study was to establish and introduce a database of immersive video clips that can serve as a resource for emotion induction research through VR. We sourced and tested a total of 73 video clips. Results showed that the stimuli varied reasonably well along the dimensions of valence and arousal. However, there appears to be a lack of representation for videos that are of negative valence yet highly arousing. In the IAPS and IADS, stimuli that belong to this quadrant tend to represent themes that are gory or violent, such as a victim of an attack that has his face mutilated, or a woman being held hostage with a knife to her throat. The majority of our videos are in the public domain and readily viewable on popular websites such as *Youtube* which have a strict policy on the types of content that can be uploaded. Hence, it is not surprising that stimuli of negative valence and arousal were not captured in our selection of immersive videos. Regardless, the collection of video clips (which can be found here) should serve as a good launching pad for researchers interested to examine the links between VR and emotion.

Although not a key factor of interest for this paper, we observed variance in the length of the video clips which was confounded with video content. Long video clips in our database tend to be of serious journalism content (e.g., nuclear fallout, homeless veterans, dictatorship regime) and naturally evoke negative valence. Length is a distinct factor of videos in contrast to photographs which are the standard emotional stimuli of photographs. Hence, while we experienced difficulty sourcing for long video clips that are of positive valence, future studies should examine the influence of video clip length on affective ratings.

The second objective sought to explore the relationship between observers' head movements and their emotions. We demonstrated a significant relationship between the amount of head yaw and valence ratings, which suggests that individuals who displayed greater movement of side-to-side head movement gave higher ratings of pleasure. However, the positive relationship shown here is in contrast to that presented by Won et al. (2016) who showed a significant relationship between the amount of head yaw and reported anxiety. It appears that content and context is an important differentiating factor when it comes to the effects of head movements. Participants in the former study explored their virtual environment and may have felt anxious in the presence of other virtual people. In our study, participants simply viewed the content presented to them without the need for navigation. Although no significant relationship was present between standard deviation of head yaw and arousal ratings, we found a correlation between head pitch and arousal, suggesting that people who tend to tilt their head upwards while watching immersive videos reported being more excited. This parallels research conducted by Lhommet and Marsella (2015) who compiled data from various studies on head positions and emotion states and showed that tilting the head up corresponds to feelings of excitement such as surprise and fear. The links between head movement and emotion are important findings and deserves further investigation.

One thing of note is the small effect sizes shown in our study (adjusted *R*^2^ = 0.05). While we tried our best to balance efficient data collection and managing participant fatigue, some participants may not be used to watching VR clips at length and may have felt uncomfortable or distressed without overtly expressing it. This may have influenced their ratings for VR clips toward the end of their study session, and may explain the small effect size. Future studies can explore when participant fatigue is likely to take place and adjust the viewing duration accordingly to minimize the impact on participant ratings.

Self-perception theory posits that people determine their attitudes based on their behavior (Bem, 1972). Future research can explore whether tasking participants to direct their head in certain directions or movements can lead to changes in their affect or attitudes. For example, imagine placing a participant in a virtual garden filled with colorful flowers and lush greenery. Since our study shows a positive link between amount of head yaw and valence ratings, will participants tasked to keep their gaze on a butterfly fluttering around them (therefore increasing the amount of head movement) lead to stronger valence compared to those who see a stationary butterfly resting on a flower? Results from this and similar studies can possibly aid in the development of virtual environments that assist patients undergoing technology-assisted therapy.

Our study examined the rotational head movements enacted by participants as they watched the video clips. Participants in our study sat on a swivel chair, which allowed them to swing around to have a full surround view of the immersive video. Future studies can incorporate translational head movements, which refers to movements that operate horizontally, laterally and vertically (x-, y-, and z- axes). This can exist through allowing participants to sit, stand or walk freely, or even program depth field elements into the immersive videos and seeing how participants' rotational and translational head movements correlate with their affect. Exploring the effects of the added degrees of freedom will contribute to a deeper understanding on the connection between head movements and emotions.

## Ethics statement

This study was carried out in accordance with the recommendations of the Human Research Protection Program, Stanford University Administrative Panel on Human Subjects in Non-Medical Research with written informed consent from all subjects. All subjects gave written informed consent in accordance with the Declaration of Helsinki. The protocol was approved by the Stanford University Administrative Panel on Human Subjects in Non-Medical Research.

## Author contributions

The authors worked as a team and made contributions throughout. BL and JB conceptualized and conducted the study. AP contributed in the sourcing and shortlisting of immersive VR clips and in revising the manuscript. WG and LW acted as domain consultants for the subject and contributed in writing and revisions.

### Conflict of interest statement

The authors declare that the research was conducted in the absence of any commercial or financial relationships that could be construed as a potential conflict of interest.

## Supplementary material

The Supplementary videos are available online at: http://vhil.stanford.edu/360-video-database/
